# Positive correlation between the nuclear expression of GPER and pGLI3 in prostate cancer tissues from patients with different Gleason scores

**DOI:** 10.3389/fendo.2024.1333284

**Published:** 2024-02-02

**Authors:** Cecilia Rico-Fuentes, Edgar Iván López-Pulido, Edsaúl Emilio Pérez-Guerrero, Marisol Godínez-Rubí, Julio César Villegas-Pineda, Martha Arisbeth Villanueva-Pérez, Erick Sierra-Díaz, José Sergio Zepeda-Nuño, Ana Laura Pereira-Suárez, Adrián Ramírez-de-Arellano

**Affiliations:** ^1^ Doctorado en Biociencias, Centro Universitario de los Altos, Universidad de Guadalajara, Tepatitlán de Morelos, Jalisco, Mexico; ^2^ Instituto de Investigación en Ciencias Biomédicas, Centro Universitario de Ciencias de la Salud, Universidad de Guadalajara, Guadalajara, Jalisco, Mexico; ^3^ Laboratorio de Patología Diagnóstica e Inmunohistoquimica, Centro de Investigación y Diagnóstico en Patología, Departamento de Microbiología y Patologia, Universidad de Guadalajara, Guadalajara, Jalisco, Mexico; ^4^ Departamento de Morfología, Centro Universitario de Ciencias de la Salud, Universidad de Guadalajara, Guadalajara, Jalisco, Mexico; ^5^ Departamento de Microbiología y Patología, Centro Universitario de Ciencias de la Salud, Universidad de Guadalajara, Guadalajara, Jalisco, Mexico; ^6^ Patología y Nefropatología, Centro de Diagnóstico e Investigación, Guadalajara, Jalisco, Mexico; ^7^ Departamento de Salud Pública, Centro Universitario de Ciencias de la Salud, División de Epidemiología, Unidad Médica de Alta Especialidad, Hospital de Especialidades, Centro Médico Nacional de Occidente, Universidad de Guadalajara, Guadalajara, Jalisco, Mexico

**Keywords:** prostate cancer, GPER, GLI1, GLI3, hedgehog, prognostic categories

## Abstract

Prostate cancer (PCa) is the most prevalent cause of death in the male population worldwide. The G Protein-Coupled Estrogen Receptor (GPER) has been gaining relevance in the development of PCa. Hedgehog (Hh) pathway activation is associated with aggressiveness, metastasis, and relapse in PCa patients. To date, no studies have evaluated the crosstalk between the GPER and the Hh pathway along different group grades in PCa. We conducted an analysis of paraffin-embedded tissues derived from patients with different prognostic grade of PCa using immunohistochemistry. Expression and correlation between GPER and glioma associated oncogene homologue (GLI) transcriptional factors in the parenchyma and stroma of PCa tumors were evaluated. Our results indicate that GPER is highly expressed in the nucleus and increases with higher grade groups. Additionally, GPER’s expression correlates with pGLI3 nuclear expression across different grade groups in PCa tissues; however, whether the receptor induces the activation of GLI transcriptional factors, or the latter modulate the expression of GPER is yet to be discovered, as well as the functional consequence of this correlation.

## 1 Introduction

Prostate cancer (PCa) is the most common non-skin malignancy in men and the fifth leading cause of cancer-related mortality worldwide (1). Even though the current treatments are effective, the molecular mechanisms related to metastasis, relapse, and treatment resistance are not fully understood. Elucidating these mechanisms would improve treatments and increase the therapeutic successes among PCa patients. For this reason, it is essential to focus on the molecules associated with this disease and the signaling pathways activated by them. Estrogens are critical hormones that regulate the development of hormone-sensitive tumors and growth disorders (2).

One of the hormonal receptors involved in PCa progression is the G protein-coupled estrogen receptor (GPER), an estradiol-activated receptor which has recently gained relevance in carcinogenesis because it promotes cell migration and invasion (3). Its role in cancer is still controversial; according to some authors GPER is protumoral (4), while others propose it as antitumoral (5), this suggests that the microenvironment might determine the effects and outcome of GPER functions. Indeed, this receptor acts through non-genomic signaling pathways related to cancer, such as mitogen-activated protein kinase (MAPK) (6), phosphoinositide 3-kinase (PI3K)/AKT (7), and hedgehog (Hh) pathway (8) in a cell-dependent manner.

Hh pathway is involved in wound healing, tissue regeneration and cell homeostasis maintenance in adults, its dysregulation is associated with cancer (9–11). Recently, a possible correlation has been suggested between high GPER expression and its overactivation of Hh signals increasing invasiveness and metastatic potential in triple-negative breast cancer (12); however, in PCa disease it has not been elucidated yet. These alterations are mainly mediated by glioma-associated oncogene homologue (GLI) transcriptional factors, which can be activated in a canonical (10) or non-canonical manner (9). GLI1 is a strong activator, whereas GLI3 has an activator/repressor domain, allowing it to act depending on the cellular context (13). Crosstalk with other pathways such as MAPK and PI3K can also activate GLI factors in a non-canonical manner (14). Therefore, GPER and activation of GLI factors probably contribute to the progression of PCa.

Amongst the GLI factors family, GLI1 and GLI3 are essential in various malignancies because they modulate cell self-renewal (15). GLI1 is involved metastatic and hormone refractory PCa (16) whilst GLI3 can either directly activate or repress target genes, including *Ptch*, *Cyclin D*, and *GLI1* (17) and its hyperactivation promotes growth and migration under depletion of androgens (18). Because the regulated expression of GLI1 and GLI3 is such a fundamental process and since dysregulation of such factors by GPER can contribute to the development and progression of PCa, it is necessary to focus on the underlying carcinogenic processes.

GLI factors are susceptible to posttranslational modifications such as phosphorylation, a phenomenon that modulates many intracellular pathways usually through crosstalk between GPER and EGFR (19). This variation between total and phosphorylated molecules leads to the upregulation of transcription genes involved in cancer invasiveness (20). However, this regulation has yet to be fully understood in PCa disease. The latter highlights the relevance to understand the phosphorylation status of GLI factors in the context of PCa.

Currently, there is a lack of information regarding the regulation that GPER exerts over GLI transcriptional factors in the context of cancer. A report suggests that an increase expression of GPER correlates with decreased nuclear GLI1 expression and unfavorable prognosis in breast cancer (21). To date, there is no evidence for a possible regulation of GLI3 by GPER. Thus, further research on the interaction between GPER and GLI transcriptional factors is needed to fully understand the mechanism involved.

Therefore, this work aimed to evaluate whether the expression of GPER in parenchyma and stromal tumor cells of PCa samples is associated with the expression and phosphorylation of GLI transcriptional factors; as well as to analyze if the expression of such factors changes according to the prognostic categories (grade groups).

## 2 Materials and methods

### 2.1 Tissue samples

We studied paraffin-embedded tissues derived from patients with different prognostic grade of PCa and that had been examined and archived at the Pathology and Nephropathology, Diagnosis and Research Center. Upon verification of the diagnosis and prognostics categories (group grades), the samples were transported to our laboratory and classified in four grade groups labelled 2,3,4, and 5 as follows: GS 7 (3 + 4) (N=9), GS 7 (4 + 3) (N=18), GS 8 (4 + 4) (N=11) and GS 9 (4 + 5) (N=15). Immunohistochemistry was performed at the Diagnostic Pathology and Immunohistochemistry laboratory at the University of Guadalajara, Mexico.

### 2.2 Automated immunohistochemistry

Formalin-fixed paraffin-embedded blocks of tissue samples were cut into 5µm sections, which were then immunostained with an automated BOND equipment (Leica Biosystems). A rabbit polyclonal primary antibody against GPER from Abcam (Cat. ab41565, Cambridge, UK) was used at a 1:200 dilution, followed by 15 minutes of incubation with EDTA buffer. GLI1 and pGLI1 from Santa Cruz (Cat.sc515781) and Biorbyt (Cat.orb503729), respectively, were used at a 1:100 dilution, followed by an incubation step with EDTA buffer for 15 minutes for GLI and 30 minutes for pGLI1. Finally, GLI3 from Santa Cruz (Cat.sc74478) and pGLI3 from Affinity Biosciences (Cat. AF7449) were used at a 1:100 dilution with 30 minutes of incubation with EDTA buffer and 30 minutes of incubation with citrate buffer, respectively. Diamond diluent from Cell Marque™ Tissue Diagnostics (Cat. 938B-05) and a DAB kit from Leica Biosystems, (Cat. DS9800) were used as antibody buffer and for detection, respectively.

The immunohistochemical analysis was performed with and Axioskop 2 plus light microscope (Carl Zeiss, Germany) coupled to a digital camera Coolsnap (Photometrics, Tucson, USA). Images were documented with Aperio LV1 scanner by Leica Biosystems and the percentage of cell positive analysis was made using Qupath version 0.2.3 software with an image type of brightfield (H-DAB). The analyses focused onto the nuclear and cytoplasmic staining and were performed using cell analysis and positive cell detection with 40x magnification. Measurements were made in parenchyma and stroma of tumor tissues.

The images were analyzed in brightfield (H-DAB) using the Qupath version 0.2.3 software. Initially, the image was adjusted to a scale bar of 80μm or 40X magnification, then the tissue grid was displayed and the area of interest was selected. After that, measure is proceeded by the next sequence: analyze/cell detection/positive cell detection. At this point, intensity threshold parameters are showed and allows you to evaluate different cell compartment with +1, +2 and +3 intensities. The data is provided by the program and recorded in an excel file for analyzed it in a statistical package.

### 2.3 Statistical analysis

We used the R version 4.1.2. software (22). Comparison of percentage of positive cells between prognostic categories and nuclear or cytoplasmatic expression were performed with two-way ANOVA followed by Bonferroni correction as a *post hoc*. Differences were considered significant at p-value < 0.05.

## 3 Results

### 3.1 GPER´s expression increases in higher grade groups in prostate tissue samples

**Figure 1:**
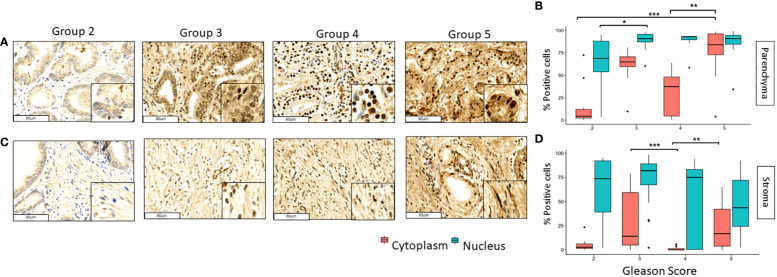
Expression of GPER in tumoral and stromal prostate tissue in different grade groups. Representative images of GPER in the parenchyma **(A, B)** and stromal **(C, D)** PCa tumor tissues by immunohistochemistry with HE staining (x200). Scale bar 80 µm. *=p<0.05, **=p<0.01, ***=p<0.001.

The expression of GPER was observed in tumoral and stromal prostate tissues, and its location in both cases predominated in the nucleus; however, it was also evidenced to a lesser extent in the cytoplasm (**Figures 1A, C**). In PCa tissues, the nuclear expression increased from grade groups 2 to 3 (*p*<0.05) and remained high in grade groups 4 and 5 samples. Likewise, GPER´s cytoplasmic expression increased significantly from grade groups 2 to 5 (*p*<0.001) and from grade groups 4 to 5 (*p*<0.01) in tumoral tissue (**Figure 1B**). In the stromal tissue, the nuclear expression in all our groups was observed and it did not change significantly throughout the different grade groups (**Figure 1C**); however, the cytoplasmic expression decreased considerable from grade groups 3 to 4 (p<0.001) while it increased from grade groups 4 to 5 (*p*<0.01) (**Figure 1D**).

### 3.2 GLI1 and pGLI1 expression predominates regardless of grades groups

**Figure 2:**
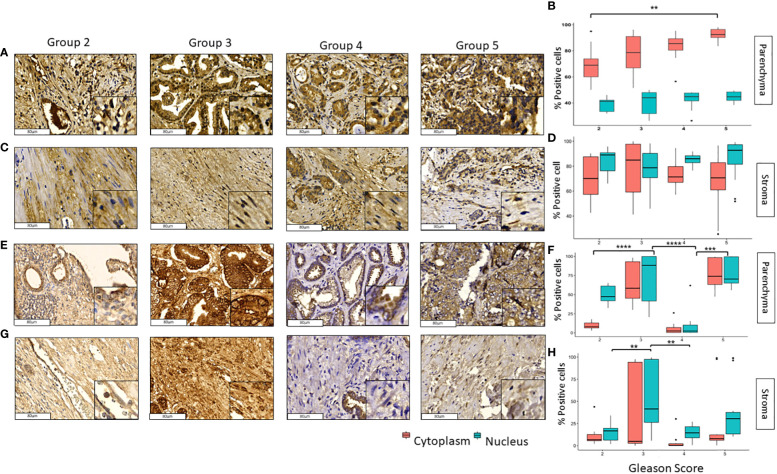
Expression of GLI1 and pGLI1 in tumoral and stromal prostate tissue in different grade groups. Representative images of GLI1 in the parenchyma **(A, B)** and stromal **(C, D)** tissues and pGLI1 in the parenchyma **(E, F)** and stromal **(G, H)** PCa tumor tissues by immunohistochemistry with HE staining (x200). Scale bar 80 µm. *=p<0.05, **=p<0.01, ***=p<0.001, ****=p<0.0001.

GLI1 and pGLI1 were expressed in the nucleus and cytoplasm in both prostate tumor and stromal tissue (**Figure 2**). GLI1 was mainly expressed in the cytoplasm of tumoral cells, significant differences were found between grade 2 vs 5 in nuclei and cytoplasm of (p<0.05) and p<0.01) respectively (**Figures 2A, B**). In stromal tissue, GLI1 was expressed in both cellular compartments with no significant changes along the different groups (**Figures 2C, D**).

In the case of pGLI1 expression did not follow a pattern either across the grade groups or across tissue type or cellular localization. In tumoral tissues, pGLI1 was mainly expressed in grade groups 3 and 5 in both nucleus and cytoplasm; however, in grade groups 4 its expression was almost null and differed significantly when compared with grade groups 3 and 5 (*p*<0.0001 and p<0.001) respectively (**Figures 2E, F**). In stromal tissue, pGLI1 was mainly expressed in the nucleus of Gleason score 3 patients. Significant differences were found between grade groups 2 vs 3 (*p*<0.01) and 3 vs 4 (*p*<0.01) in the nuclear expression of pGLI1. No significative changes were observed in the pGLI1´s cytoplasmic expression in the tumoral tissue (**Figures 2G, H**).

### 3.3 High nuclear expression in pGLI3 as grade group increase in prostate tumor tissues

**Figure 3:**
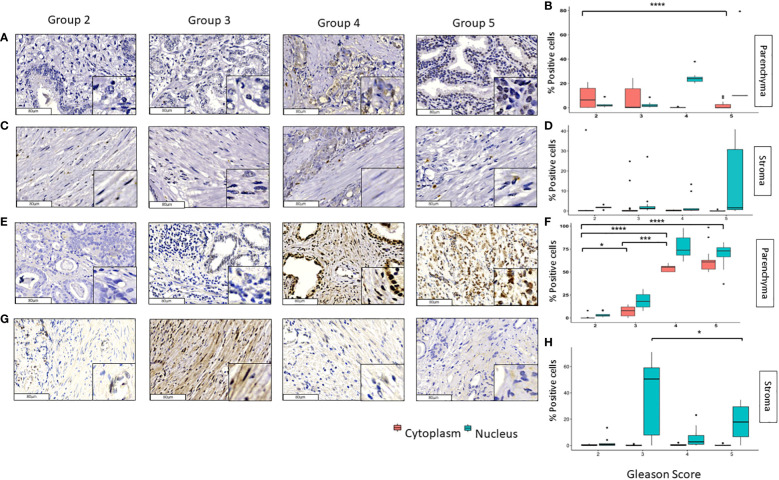
Expression of GLI3 and pGLI3 in tumoral and stromal prostate tissue in different grade groups. Representative images of GLI3 in the parenchyma **(A, B)** and stromal **(C, D)** PCa tumor tissues and pGLI3 in the tumoral **(E, F)** and stromal **(G, H)** PCa tumor tissues by immunohistochemistry with HE staining (x200). Scale bar 80 µm. *=p<0.05, **=p< 0.01, ***=p<0.001, ****=p<0.0001.

In contrast to the other transcription factors, GLI3 and pGLI3 were lightly observed in the tumoral and stromal tissues (**Figure 3**). In tumoral tissue, the cytoplasmic expression of GLI3 decreases considerably from grade groups 2 to 5 and 3 to 5 (*p*<0.0001). The nuclear expression of GLI3 in these samples was low, and no significant changes were observed in the different groups (**Figures 3A, B**). As for the stromal tissue, GLI3 was absent in practically all group grades with non-significant *p* values among them (**Figures 3C, D**). Interestingly, nuclear pGLI3 increased significantly from grade groups 2 vs 3 (*p*<0.001) and 2 vs 4,5 (*p*<0.0001) in addition the cytoplasmic expression also increases from grades 2 vs 4 and 3 vs 4 (*p*<0.0001) (**Figures 3E, F**). Finally, the expression of pGLI3 decreases significantly from grade groups 3 to 5 (p<0.05) in stromal tissue (**Figures 3G, H**).

### 3.4 GPER positively correlates with pGLI3 factors in tumor tissues

**Figure 4:**
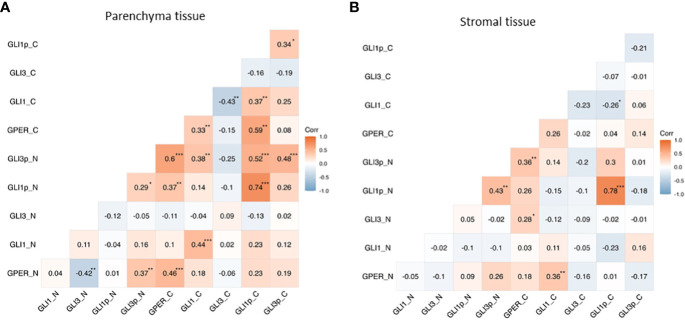
Correlation of GPER and GLI transcriptional factors in nuclear (N) and cytoplasmic (C) expression in the parenchyma and stromal PCa tissues. Heatmaps showing the correlation between the expression of GPER and GLI1, pGLI1, GLI3, and pGLI3 in parenchyma **(A)** and stromal **(B)** tissue. *p<0.05, **p<0.01, ***p<0.001.

A Pearson correlation revealed that in the tumoral tissue nuclear GPER positively correlates with nuclear pGLI3 (*p*<0.01) and negatively correlated with nuclear GLI3 (*p*<0.01). While cytoplasmic GPER positively correlates with cytoplasmic GLI1 and pGLI1 (*p*<0.01). Furthermore, there was a positive correlation in the expression of GPER vs pGLI1 and pGLI3 in the nuclear localization with a *p*<0.01 and *p*<0.001 respectively. Of note, these GLI transcriptional factors (pGLI1 and pGLI3) also correlated with each other (*p*<0.05). As for the cytoplasmic expression, we found that GLI1 was negatively correlated with GLI3 (*p*<0.01) whereas nuclear pGLI3 had a positive correlation with pGLI1 (*p*<0.001) (**Figure 4A**).

In stromal tissue, there was a positive correlation between nuclear GPER´s expression and GLI1´s in the cytoplasm (*p*<0.01). Also, the cytoplasmic expression of GPER was positively correlated with the nuclear pGLI3 (*p*<0.01). Moreover, nuclear pGLI1 and pGLI3 were positively correlated (*p*<0.01) (**Figure 4B**).

## 4 Discussion

PCa is the most common malignancy that impacts men’s health, generating a pathological and molecular challenge in the understanding of the disease. GPER has been identified in parenchymal and stromal cells showing a dual role in proliferation depending on specific site of expression in reproductive tissues and regulates protumorigenic events by the activation of several signaling pathways (23). Hh pathway is a key signaling pathway involved in stemness and associated to relapse and bad prognosis (11) Not much is known about GPER and its regulation of Hh transcriptional factors, nevertheless, this receptor can trigger signaling cascades initiated in plasma membrane (24) leading to a pro-tumorigenic environment that promote angiogenesis (25); however, there is a limited information about biological significance of GPER and relation with Hh pathways in PCa tissue. In this study, we evaluated the expression of GPER and its correlation of GLI factor involved in PCa tissues with different prognostic groups.

Our first result indicates that GPER´s expression remains in nuclear and increase in cytoplasmic compartments as grade groups proceed in tumor parenchyma, this phenomenon is discordant with Rago et al. studies where cytoplasmic GPER expression was highly observed in benign and decreases in prostate intraepithelial neoplasia lesions from Italian patients (26). A possible explanation might be the antibody used in both studies. Different epitopes are recognized by the antibodies, the antibody used in Rago´s research recognized extracellular domain (26) while the one we used can detect a protein ubiquitously expressed 27). Nuclear GPER was also reported by Marco Pupo et al. in breast cancer-associated fibroblasts, they suggest that GPER translocate through an importin-dependent mechanism and upregulated target genes like c-fos and CTGF induce by estrogens (28). Our results can lead to generate future perspectives performing cell fractions, to determine gene expression that GPER is activated in PCa tissues.

GPER may modulate the expression of genes (23) associated to the progression and development of PCa; however, further studies are needed to prove this hypothesis. Thus, targeting this receptor will provide a way to progress to a more precise approach to hormone-related disease detection and management.

Another fact was the presence of GPER expression in cytoplasm in tumoral and stroma PCa tissues, this remains as grade group proceed which agrees with Rago (26) and T Yu (29) studies. Several reports evidence GPER’s modulation on clinical outcome, for instance Rago mentions that low expression of GPER is associated with high expression of pAKT and pCREB involved in cancer relapses (26); on the other hand, Yu et al. published that GPER confers multidrug resistance in CAFs through a cAMP/PKA/CREB dependent manner (29), therefore the expression of GPER is crucial to determine the level of aggressiveness in PCa tissues. Further analyses are required to demonstrate this fact in PCa.

In our results GLI1 full length (GLI1) was expressed mainly in cytoplasmic location in stroma and tumor tissue regardless of the grade group. This agrees with the observations made by Xing Liu et al., research which suggest that suppressor of fused protein (SUFU) has a negative regulation of Hh/GLI signaling activity, which arrests GLI1 proteins in cytoplasm and prevents their translocation to the nucleus (30). Therefore, SUFU protein affects transcription activity in cytoplasmic location (31). To date, still no evidence about correlation or interaction among GPER/SUFU/GLI1 in participation of diseases.

On the other hand, we found GLI1 phosphorylated (pGLI1) in tumor and stroma tissues where the expression was observed mainly in Gleason 7 (4 + 3) and 9 (4 + 5) (grade groups 3 and 5 respectively). Currently there are no reports about the behavior regarding presence and absence of pGLI1 along the different grade groups in prostate cancer tissues; however, pGLI1 is known to activate MAPK-ERK1/2 signaling in a SMO-independent manner that can be induced by stimulation of VEGFA secreted by cancer stromal cells in a paracrine manner in lung adenocarcinoma (13). Therefore, more studies are required to elucidate the importance of GPER/pGLI1 in tumor and stroma tissues.

Finally, we evaluated the expression of GLI3 full length (GLI3) in our samples. Slight expression was found in both compartments throughout grade groups. This was also observed by Diana Trnski et al., concluding that the processing of GLI3 into repressor transcriptional form is due to downregulation of GSK3β therefore cell proliferation decreases in colon cancer (30). Also GLI3 is degraded by proteasome induction of speckle-type POZ protein (SPO) (32) during CRCP androgen deprivation (18). Therefore, SPO and GSK3β are probably involved in downregulation or degradation of GLI3 and promotes tumor aggressiveness in PCa tissues.

Nevertheless, pGLI3 was observed in the nucleus of tumor parenchyma cells and, interestingly, its expression increased as the prognostic grade progresses. A few reports mention that phosphorylation of this transcriptional factor regulates positively (10) and negatively (33) in cancer, due to transactivation domain and the presence of PKA, GSK3β, and βTrCP. Also, it has been reported to directly interact with androgen receptor (AR), leading to its nuclear translocation (9) and consequently activate target genes, such as *cyclinD1* and *Fgf15* (34).

Due to the latter observations, we decided to evaluate if there was a correlation between the expression of the evaluated molecules. We observed a positive correlation among nuclear and cytoplasmic GPER with pGLI3 in the nuclear localization. Currently, there is no evidence on the regulation that GPER exerts in these compartments, and it needs to be elucidated in PCa disease. GPER is known to modulate several signaling pathways, PI3K/Akt among them, and the crosstalk between this pathway and GLI activation has previously been reported (14). This could stimulate importin-dependent mechanisms and modulate GSK3β, enabling GPER and pGLI3 translocate into the nucleus. Therefore, it would be an important perspective to evaluate the genes activated by GPER and pGLI3 in the PCa disease.

As complementary data, GLI1 and GLI3 had a negative correlation in their cytoplasmic expression in tumoral tissue. Both factors can interact and modulate each other’s activity in cancer cells (9). In PCa it is known that GLI factors are upregulated in presence or absence of androgen, western blots showed that in the presence of AR, GLI3 expression increased in LNCaP cells; however, in the absence of AR GLI1 is predominated. This suggests that hormonal factors and crosstalk from oncogenic signaling pathways affects GLI transcriptional activity in PCa disease (9, 13). This fact has been linked to several non-canonical oncogenic growth signals and demonstrates the contribution of GLI1in differentiation during cancer development (35).

## 5 Conclusion

GPER has been involved in the regulatory mechanisms in prostate cancer cells; however, the mechanisms underlying these effects are still not fully understood. In this work, the presence of GPER in the nucleus was observed, and a positive correlation with pGLI3 transcriptional factor was established. Interestingly this correlation is maintained in the different prognostic groups. Whether GPER regulates pGLI3 or vice versa is still yet to be discovered, and further analysis should be conducted to solve this question. In a future direction, it would be interesting to describe the genes these molecules activate; this would light up the path in understanding the antitumoral GPER actions.

## Data availability statement

The raw data supporting the conclusions of this article will be made available by the authors, without undue reservation.

## Ethics statement

The studies involving humans were approved by Ethics Committee of the University Center for Health Sciences. The studies were conducted in accordance with the local legislation and institutional requirements. The present investigation was approved by the Ethics Committee of the University Center for Health Sciences (Opinion No. CI-01719), where exemption from informed consent was considered, based on the guidelines stipulated in CIOMS Guideline 10. The following points were taken into consideration: the research could not be conducted without the exemption from informed consent, considering that our retrospective study was based on the collection of remaining samples from pathological diagnosis; there is no risk to the participants in the study, as personal data information was dissociated, and patient identification is not possible. In addition, this research holds significant social and scientific value for the Mexican population under study.

## Author contributions

CR-F: Investigation, Writing – original draft. EL-P: Investigation, Writing – review & editing. EP: Formal analysis, Methodology, Writing – review & editing. MG: Formal analysis, Investigation, Methodology, Resources, Supervision, Writing – review & editing. JV-P: Investigation, Methodology, Visualization, Writing – review & editing. MV-P: Methodology, Resources, Writing – review & editing. ES: Resources, Supervision, Visualization, Writing – review & editing. SZ: Methodology, Supervision, Writing – review & editing. AP-S: Conceptualization, Investigation, Resources, Supervision, Visualization, Writing – review & editing. AR-d-A: Conceptualization, Funding acquisition, Investigation, Project administration, Resources, Supervision, Visualization, Writing – review & editing.
